# Draft Genomes of 83 *Vibrio* Isolates from Fresh Canadian Mollusks

**DOI:** 10.1128/mra.00749-22

**Published:** 2022-11-07

**Authors:** Januana S. Teixeira, Forest Dussault, Emily Hoover, Julie A. Shay, Kelly Weedmark, Swapan K. Banerjee

**Affiliations:** a Bureau of Microbial Hazards, Health Canada, Ottawa, Ontario, Canada; b Bureau of Food Surveillance and Science Integration, Health Canada, Ottawa, Ontario, Canada; Indiana University, Bloomington

## Abstract

A subset of *Vibrio* spp. isolated from fresh Canadian mollusks (2014 to 2018) were selected for sequencing based on antimicrobial resistance profiles. The resulting *de novo* draft genomes include 38 Vibrio alginolyticus, 32 V. diabolicus, 10 V. parahaemolyticus, 1 V. cholerae, 1 V. ordalii, and 1 *Vibrio* sp. isolate.

## ANNOUNCEMENT

*Vibrio* spp. are a diverse group of Gram-negative bacteria ubiquitously present in marine environments (1). Consumption of raw or undercooked mollusks such as oysters, mussels, and clams contaminated with pathogenic *Vibrio* spp. can lead to severe gastrointestinal infections in humans (1, 2). In Canada’s maritime provinces, the risk of foodborne illness from *Vibrio* spp. increases during the warmer months, when these pathogens are most abundant in the marine environment (3, 4). Antimicrobial resistance (AMR) in *Vibrio* spp. has been an increasing concern due to horizontal gene transfer and exposure to AMR bacteria (1, 4).

Mollusk samples were collected during the harvest season (May to October) of 2014 through 2018. Freshly harvested samples (*n* = 47) were immediately stored on ice, shipped, and processed within 48 h of harvest as described elsewhere (5). Briefly, 10 to 20 shucked mollusks per sample were homogenized in a blender; 50 g of homogenate was mixed with 450 mL alkaline peptone water (1% Bacto peptone, 2% NaCl; pH 8.5) and equilibrated for 1 h at 21°C before overnight enrichment at 35°C. Selective agars (Thiosulfate-Citrate-Bile Salts-Sucrose, CHROMagar, and mCC10 or CC400) were used for both pre-enrichment (100 μL) and postenrichment (10 μL) plating. After 35°C overnight incubation, presumptive *Vibrio* colonies were grown at 35°C overnight on tryptic soy agar with 2% NaCl (TSA-2N) (Difco BD, Franklin Lakes, NJ, USA) for molecular/biochemical *Vibrio* confirmation (5) and for storage as −80°C stocks (one 10-μL loopful per Microbank vial) (VWR, Mississauga, Ontario, Canada). AMR phenotype relative to 23 antibiotics (Oxoid Ltd., Basingstoke, UK) was determined for isolates using the Kirby-Bauer disk diffusion method and published guidelines (6–8); a subset of 83 isolates were selected for sequencing (Table 1).

**TABLE 1:** Metadata and genome assembly metrics for 83 *Vibrio* isolates from fresh mollusks harvested in Canada from 2014 to 2018 (NCBI BioProject PRJNA839006)

Isolate	Sample	Yr	Sampling location^*a*^	AMR profile^*b*^	Taxonomy ID (Mash)	Genome size (bp)	% GC	Coverage (×)	No. of reads	No. of contigs	*N*_50_ (bp)	No. of genes	Accession no.		
													BioSample	GenBank	SRA
HS-1-2	Clam	2014	Smith's Cove, NS	AMP, PIP, KF	V. alginolyticus	5,279,810	44.5	81	1,742,704	58	246,583	4,929	SAMN28494979	JAMQAF000000000	SRR19505466
HS-3-10	Oyster	2014	Eel Lake, NS	AMP, PIP, KF	V. alginolyticus	5,296,870	44.5	36	768,012	70	176,384	4,886	SAMN28494980	JAMQAE000000000	SRR19505465
HS-6-2	Clam	2014	Gaspé, QC	AMP, PIP, KF	V. alginolyticus	5,181,727	44.6	53	1,075,070	58	231,692	4,812	SAMN28494981	JAMQAD000000000	SRR19505454
HS-6-3	Clam	2014	Gaspé, QC	AMP, KF, S	V. parahaemolyticus	5,035,607	45.4	28	586,672	79	137,596	4,638	SAMN28494982	JAMQAC000000000	SRR19505443
HS-7-2	Clam	2014	Smith's Cove, NS	AMP, KF, S, E	V. parahaemolyticus	4,942,691	45.4	39	803,332	60	194,100	4,562	SAMN28494983	JAMQAB000000000	SRR19505432
HS-9-7	Clam	2014	Gaspé, QC	AMP, PIP, KF, S, PB, E	V. alginolyticus	5,247,640	44.7	41	860,084	89	124,394	4,808	SAMN28494984	JAMQAA000000000	SRR19505421
HS-9-13	Clam	2014	Gaspé, QC	AMP, PIP, KF (E, OT)	V. alginolyticus	5,170,302	44.6	55	1,283,154	62	231,641	4,751	SAMN28494985	JAMPZZ000000000	SRR19505410
HS-11-6A	Oyster	2014	Eel Lake, NS	AMP, PIP, KF	V. alginolyticus	5,167,959	44.6	90	1,787,684	81	151,557	4,801	SAMN28494986	JAMPZY000000000	SRR19505399
HS-14-8	Oyster	2014	Eel Lake, NS	AMP, KF, K	V. alginolyticus	5,209,924	44.6	36	763,220	77	112,952	4,761	SAMN28494987	JAMPZX000000000	SRR19505388
HS-21-9A	Oyster	2014	Eel Lake, NS	AMP, PIP, KF	V. alginolyticus	5,190,156	44.6	23	540,284	153	73,880	4,784	SAMN28494988	JAMPZW000000000	SRR19505384
HS-23-8	Clam	2014	Gaspé, QC	AMP, PIP, KF	V. alginolyticus	5,074,093	44.5	109	2,204,816	44	281,992	4,697	SAMN28494989	JAMPZV000000000	SRR19505464
HS-25-4	Clam	2015	Smith's Cove, NS	AMP, PIP, KF	V. alginolyticus	5,253,555	44.5	41	914,232	59	225,898	4,842	SAMN28494990	JAMPZU000000000	SRR19505463
HS-26-7	Clam	2015	Gaspé, QC	AMP (KF, E, ENO, OT)	*V. diabolicus*	5,276,346	44.8	55	1,197,486	47	402,198	4,856	SAMN28494991	JAMQRO000000000	SRR19505462
HS-27-4	Clam	2015	Smith's Cove, NS	AMP, PIP, KF	*V. diabolicus*	5,159,751	44.8	128	2,580,938	45	367,116	4,715	SAMN28494992	JAMQRN000000000	SRR19505461
HS-28-5	Clam	2015	Gaspé, QC	AMP, KF, OX	V. alginolyticus	5,394,800	44.6	66	1,429,102	64	243,159	4,950	SAMN28494993	JAMPZT000000000	SRR19505460
HS-29-8	Clam	2015	Smith's Cove, NS	AMP, PIP, KF, SF, OX	*V. diabolicus*	5,148,699	44.7	44	915,460	58	346347	4819	SAMN28494994	JAMQRM000000000	SRR19505459
HS-30-8	Clam	2015	Gaspé, QC	AMP, PIP, KF, SF	V. alginolyticus	5,173,808	44.6	50	1,095,278	46	248,013	4,775	SAMN28494997	JAMPZQ000000000	SRR19505456
HS-30-11	Clam	2015	Gaspé, QC	AMP, PIP, KF, SF	V. alginolyticus	5,171,704	44.6	66	1,556,322	38	455,773	4,760	SAMN28494995	JAMPZS000000000	SRR19505458
HS-30-16	Clam	2015	Gaspé, QC	AMP, PIP, KF, SF	V. alginolyticus	5,135,833	44.7	52	980,026	142	73,597	4,727	SAMN28494996	JAMPZR000000000	SRR19505457
HS-31-7	Clam	2015	Smith's Cove, NS	AMP, PIP, SF, OX	*V. diabolicus*	5,073,771	44.7	77	1,542,468	59	233,837	4,758	SAMN28494998	JAMQRL000000000	SRR19505455
HS-31-9	Clam	2015	Smith's Cove, NS	AMP, PIP, SF, OX	*V. diabolicus*	5,153,063	44.7	72	1,475,488	59	220,846	4,835	SAMN28494999	JAMQRK000000000	SRR19505453
HS-32-1	Clam	2015	Gaspé, QC	AMP, KF (IPM, E, OT)	*V. ordalii*	4,016,157	44.6	165	2,608,402	85	368,741	3,667	SAMN28495000	JAMQRJ000000000	SRR19505452
HS-32-2	Clam	2015	Gaspé, QC	AMP, PIP, KF	V. alginolyticus	5,250,985	44.6	46	1,007,826	40	330,647	4,838	SAMN28495001	JAMPZP000000000	SRR19505451
HS-32-5	Clam	2015	Gaspé, QC	AMP, KF, SF, OX	*V. diabolicus*	4,942,772	45.0	43	729,456	265	35,098	4,577	SAMN28495002	JAMQRI000000000	SRR19505450
HS-32-8	Clam	2015	Gaspé, QC	AMP, PIP, SF, OX	*V. diabolicus*	5,247,686	44.8	38	993,638	59	257,008	4,895	SAMN28495003	JAMQRH000000000	SRR19505449
HS-32-9	Clam	2015	Gaspé, QC	AMP, KF, OX	*V. diabolicus*	5,068,562	44.9	44	999,746	57	206,822	4,679	SAMN28495004	JAMQRG000000000	SRR19505448
HS-34-5	Clam	2015	Gaspé, QC	AMP, PIP, KF	V. alginolyticus	5,302,700	44.5	28	641,034	116	103,790	4,877	SAMN28495005	JAMPZO000000000	SRR19505447
HS-34-6	Clam	2015	Gaspé, QC	AMP, PIP, KF, SF	V. alginolyticus	5,314,580	44.5	75	1,587,218	48	385,246	4,977	SAMN28495006	JAMPZN000000000	SRR19505446
HS-35-11	Oyster	2015	Eel Lake, NS	AMP, PIP, OX	V. alginolyticus	5,236,535	44.6	53	1,212,078	64	198,011	4,813	SAMN28495007	JAMPZM000000000	SRR19505445
HS-36-4	Clam	2015	NS	AMP, KF, S	V. parahaemolyticus	5,011,762	45.3	62	1,217,808	58	228,589	4,628	SAMN28495008	JAMPZL000000000	SRR19505444
HS-36-5	Clam	2015	NS	AMP, PIP, OX	*V. diabolicus*	5,197,981	44.7	139	2,756,570	49	552,486	4,887	SAMN28495009	JAMQRF000000000	SRR19505442
HS-37-7	Oyster	2015	Eel Lake, NS	AMP, KF, SF, OX	*V. diabolicus*	5,347,404	44.7	107	2,502,662	50	344,809	4,980	SAMN28495010	JAMQRE000000000	SRR19505441
HS-39-7	Oyster	2015	Eel Lake, NS	AMP, PIP, KF	V. alginolyticus	5,112,278	44.5	55	1,207,080	47	254,573	4,686	SAMN28495011	JAMPZK000000000	SRR19505440
HS-40-3	Clam	2015	Smith's Cove, NS	AMP, PIP, KF, OX	*V. diabolicus*	5,170,365	44.8	76	1,525,590	44	301,603	4,779	SAMN28495012	JAMQRD000000000	SRR19505439
HS-41-4	Clam	2015	Gaspé, QC	AMP, PIP, KF, SF	V. alginolyticus	5,230,739	44.6	73	1,574,962	61	442,486	4,837	SAMN28495013	JAMPZJ000000000	SRR19505438
HS-41-6	Clam	2015	Gaspé, QC	AMP, PIP, S	V. alginolyticus	5,247,006	44.5	61	1,431,306	46	338,496	4,848	SAMN28495014	JAMPZI000000000	SRR19505437
HS-43-1	Oyster	2016	Metcalfe Bay, BC	AMP, PIP, OX	*V. diabolicus*	5,321,336	44.8	58	1,347,776	61	251,182	4,930	SAMN28495015	JAMQRC000000000	SRR19505436
HS-43-2	Oyster	2016	Metcalfe Bay, BC	AMP, PIP, OX	*V. diabolicus*	5,163,393	44.8	143	3,885,228	36	446,110	4,775	SAMN28495016	JAMQRB000000000	SRR19505435
HS-43-3	Oyster	2016	Metcalfe Bay, BC	AMP, PIP, OX	*V. diabolicus*	5,251,744	44.7	110	2,612,064	41	356,719	4,861	SAMN28495017	JAMQRA000000000	SRR19505434
HS-43-5	Oyster	2016	Metcalfe Bay, BC	AMP, PIP, KF, OX	*V. diabolicus*	6,124,036	43.5	95	2,361,022	59	404,856	5,974	SAMN28495018	JAMQQZ000000000	SRR19505433
HS-43-6	Oyster	2016	Metcalfe Bay, BC	AMP, PIP, KF, OX	*V. diabolicus*	6,124,301	43.7	80	2,043,750	57	457,829	6,056	SAMN28495019	JAMQQY000000000	SRR19505431
HS-43-7A	Oyster	2016	Metcalfe Bay, BC	AMP, KF (PIP, CTX, CEF, E, ENO, OX, OT)	*V. diabolicus*	5,940,983	43.7	82	1,978,856	58	357,386	5,747	SAMN28495020	JAMQQX000000000	SRR19505430
HS-43-7B	Oyster	2016	Metcalfe Bay, BC	AMP, KF, OT	V. alginolyticus	5,368,506	44.4	25	554,486	166	85,447	5,051	SAMN28495021	JAMPZH000000000	SRR19505429
HS-46-5	Oyster	2016	Parksville, BC	AMP, PIP, KF	V. alginolyticus	5,396,729	44.5	129	3,050,086	48	433,779	5,025	SAMN28495022	JAMPZG000000000	SRR19505428
HS-47-7	Oyster	2016	Eel Lake, NS	AMP, PIP, KF, OX	*V. diabolicus*	5,008,741	44.8	30	689,282	95	115,568	4,657	SAMN28495023	JAMQQW000000000	SRR19505427
HS-50-1	Oyster	2016	Parksville, BC	AMP, PIP, KF, OX	*Vibrio* spp.	5,289,568	44.8	152	3,266,532	49	441,934	4,943	SAMN28495024	JAMPZF000000000	SRR19505426
HS-50-2	Oyster	2016	Parksville, BC	AMP, PIP, KF, SF, OX	*V. diabolicus*	5,194,668	44.8	67	1,314,048	41	436,139	4,818	SAMN28495025	JAMQQV000000000	SRR19505425
HS-50-3	Oyster	2016	Parksville, BC	AMP, PIP, KF	V. alginolyticus	5,272,228	44.5	180	3,430,338	46	237,442	4,847	SAMN28495026	JAMPZE000000000	SRR19505424
HS-50-4	Oyster	2016	Parksville, BC	AMP, PIP, KF, OX, OT	*V. diabolicus*	5,354,891	44.6	59	1,246,740	45	428,472	4,976	SAMN28495027	JAMQQU000000000	SRR19505423
HS-51-4	Clam	2016	Smith's Cove, NS	AMP, KF (CEF, E, ENO, OX, OT)	*V. diabolicus*	5,260,487	44.8	59	1,187,042	38	406,357	4,842	SAMN28495028	JAMQQT000000000	SRR19505422
HS-51-5	Clam	2016	Smith's Cove, NS	AMP (KF, E, ENO)	V. alginolyticus	5,327,807	44.5	76	1,567,682	62	233,100	4,925	SAMN28495029	JAMPZD000000000	SRR19505420
HS-52-6	Clam	2016	BC	AMP, KF, OX	*V. diabolicus*	5,197,446	44.8	178	3,344,254	49	462,455	4,820	SAMN28495030	JAMQQS000000000	SRR19505419
HS-53-2	Oyster	2016	Eel Lake, NS	AMP, KF, S	*V. diabolicus*	5,398,014	44.6	81	1,564,256	44	365,596	5,026	SAMN28495032	JAMQQR000000000	SRR19505417
HS-53-10	Oyster	2016	Eel Lake, NS	AMP, PIP, OX	V. parahaemolyticus	5,032,476	45.2	43	878,212	55	264,514	4,674	SAMN28495031	JAMPZC000000000	SRR19505418
HS-56-5	Clam	2016	Smith's Cove, NS	AMP, PIP, KF	V. alginolyticus	5,228,619	44.6	80	1,558,618	43	317,697	4,804	SAMN28495033	JAMPZB000000000	SRR19505416
HS-57-1	Oyster	2016	Parksville, BC	AMP, KF, OX	*V. diabolicus*	5,157,215	44.9	165	3,065,612	39	399,323	4,747	SAMN28495034	JAMQQQ000000000	SRR19505415
HS-57-7	Oyster	2016	Parksville, BC	AMP, PIP, KF, SF	*V. diabolicus*	5,273,448	44.7	105	1,995,998	57	408,030	4,946	SAMN28495035	JAMQQP000000000	SRR19505414
HS-57-10	Oyster	2016	Parksville, BC	AMP, PIP, KF, SF, OX	*V. diabolicus*	5,043,465	44.8	68	1,484,814	63	197,073	4,737	SAMN28495036	JAMQQO000000000	SRR19505413
HS-58-1	Clam	2016	Gaspé, QC	AMP, PIP, KF	V. alginolyticus	5,269,803	44.6	112	2,120,760	53	221,952	4,904	SAMN28495037	JAMPZA000000000	SRR19505412
HS-58-3	Clam	2016	Gaspé, QC	AMP, PIP, KF	V. alginolyticus	5,188,730	44.5	80	1,542,860	55	221,952	4,807	SAMN28495038	JAMPYZ000000000	SRR19505411
HS-59-6	Clam	2016	Smith's Cove, NS	AMP, PIP, KF, OX	*V. diabolicus*	5,101,415	44.8	76	1,477,328	34	435,153	4,704	SAMN28495039	JAMQQN000000000	SRR19505409
HS-60-3	Oyster	2016	Eel Lake, NS	AMP, PIP, KF	*V. diabolicus*	5,089,027	44.8	84	1,779,960	48	463,036	4,739	SAMN28495041	JAMQQM000000000	SRR19505407
HS-60-11	Oyster	2016	Eel Lake, NS	AMP, PIP, KF, SF, OX	V. parahaemolyticus	5,148,398	45.2	121	2,222,476	47	257,051	4,740	SAMN28495040	JAMPYY000000000	SRR19505408
HS-61-1	Clam	2016	Gaspé, QC	AMP, PIP, KF, SF	V. alginolyticus	5,262,756	44.6	53	1,044,406	51	222,598	4,839	SAMN28495042	JAMPYX000000000	SRR19505406
HS-61-11C	Clam	2016	Gaspé, QC	AMR, KF (CTX, CEF, E, ENO, OT)	V. parahaemolyticus	5,085,472	45.3	26	572,568	87	130,613	4,719	SAMN28495043	JAMPYW000000000	SRR19505405
HS-61-3	Clam	2016	Gaspé, QC	AMP, PIP, KF	V. alginolyticus	5,317,358	44.5	53	1,194,060	69	215,779	4,909	SAMN28495044	JAMPYV000000000	SRR19505404
HS-62-8	Oyster	2016	Parksville, BC	AMP, PIP, OX	*V. diabolicus*	5,070,117	44.8	104	2,346,012	48	567,202	4,756	SAMN28495045	JAMQQL000000000	SRR19505403
HS-62-9	Oyster	2016	Parksville, BC	AMP, KF, SF	V. parahaemolyticus	5,283,549	45.3	121	2,417,766	68	194,545	4,850	SAMN28495046	JAMPYU000000000	SRR19505402
HS-63-3	Mussel	2016	BC	AMP, PIP, KF	V. alginolyticus	5,202,885	44.6	76	1,575,390	47	422,893	4,764	SAMN28495047	JAMPYT000000000	SRR19505401
HS-63-7	Mussel	2016	BC	AMP, PIP, OX	*V. diabolicus*	5,191,291	44.9	138	2,632,458	44	815,460	48,20	SAMN28495048	JAMQQK000000000	SRR19505400
HS-63-8	Mussel	2016	BC	AMP, PIP, KF	V. alginolyticus	5,200,087	44.5	129	2,536,190	44	226,658	4,763	SAMN28495049	JAMPYS000000000	SRR19505398
HS-64-4	Clam	2016	Smith's Cove, NS	AMP, PIP, SF, OX	*V. diabolicus*	5,172,367	44.7	39	1,019,306	83	157,536	4,847	SAMN28495050	JAMQQJ000000000	SRR19505397
HS-64-5	Clam	2016	Smith's Cove, NS	AMP, KF, OX	*V. diabolicus*	5,113,674	44.8	97	2,130,112	45	423,139	4,670	SAMN28495051	JAMQQI000000000	SRR19505396
HS-65-4	Oyster	2016	Eel Lake, NS	AMP, PIP, KF, OX	V. alginolyticus	5,254,705	44.6	59	1,316,926	56	225,355	4,834	SAMN28495052	JAMPYR000000000	SRR19505395
HS-66-3	Oyster	2016	Metcalfe Bay, BC	AMP, PIP, KF, SF	V. alginolyticus	5,258,746	44.6	80	1,650,180	40	508,914	4,823	SAMN28495053	JAMPYQ000000000	SRR19505394
HS-66-6	Oyster	2016	Metcalfe Bay, BC	AMP, PIP, KF	V. alginolyticus	5,369,146	44.5	61	1,261,890	62	263,734	4,953	SAMN28495054	JAMPYP000000000	SRR19505393
HS-67-6	Oyster	2016	NB	AMP, PIP, KF	V. alginolyticus	5,399,019	44.5	138	2,695,004	63	325,897	5,029	SAMN28495055	JAMPYO000000000	SRR19505392
HS-67-7	Oyster	2016	NB	AMP, PIP, KF	V. parahaemolyticus	5,121,474	45.2	68	1,401,988	36	384,982	4,771	SAMN28495056	JAMPYN000000000	SRR19505391
HS-67-8	Oyster	2016	NB	AMP, PIP, KF	V. alginolyticus	5,047,512	44.6	95	1,707,472	36	435,643	4,502	SAMN28495057	JAMPYM000000000	SRR19505390
HS-69-5	Clam	2016	Gaspé, QC	AMP, PIP, KF, SF	V. alginolyticus	5,342,446	44.5	58	1,169,740	70	178,422	5,068	SAMN28495058	JAMPYL000000000	SRR19505389
HS-80-7	Oyster	2017	Eel Lake, NS	AMP (KF, CTX, E, ENO, OX)	V. parahaemolyticus	4,909,992	45.5	96	1,800,744	134	82,946	4,570	SAMN28495059	JAMPYK000000000	SRR19505387
HS-92-7	Oyster	2017	Eel Lake, NS	None	V. parahaemolyticus	5,872,986	44.2	89	1,870,694	114	123,462	5,740	SAMN28495060	JAMPYJ000000000	SRR19505386
HS-119-4	Clam	2018	Gaspé, QC	None	V. cholerae	4,120,304	47.5	65	968,146	155	83,542	3,884	SAMN28495061	JAMPYI000000000	SRR19505385

a BC, British Columbia; NB, New Brunswick; NS, Nova Scotia; QC, Québec.

b AMP, ampicillin; CEF, ceftiofur; CTX, cefotaxime; C, chloramphenicol; CIP, ciprofloxacin; DOR, doripenem; ETP, ertapenem; E, erythromycin; ENO, enrofloxacin; GN, gentamicin; IPM, imipenem; K, kanamycin; KF, cephalothin; MEM, meropenem; NOR, norfloxacin; OT, oxytetracycline; OX, oxolinic acid; PIP, piperacillin; PB, polymyxin B; S, streptomycin; SF, sulfisoxazole; SXT, sulfamethoxazole-trimethoprim; TE, tetracycline; None, no resistance. Parentheses indicate intermediate resistance.

For sequencing, frozen stocks were streaked onto TSA-2N (Difco BD, Franklin Lakes, NJ, USA) and incubated at 35°C overnight. An isolated colony was used to inoculate 5 mL tryptic soy broth with 2% NaCl (TSB-2N) at 35°C for 24 h, and DNA was extracted using the Maxwell 16 SEV cell DNA purification kit (Promega, Madison, WI, USA). Paired-end Illumina sequencing was performed using indexed Nextera XT libraries run on a MiSeq instrument (2 × 300 cycles, v3 chemistry) according to the manufacturer (Illumina, San Diego, CA, USA).

Reads were trimmed to remove adapters, quality filtered, and error corrected (BBMap v38.26 [BBDuk and Tadpole]) (https://sourceforge.net/projects/bbmap) for *de novo* assembly (SKESA v2.3, Pilon v1.22) (9), gene prediction (NCBI PGAP v6.1; best-placed reference protein set; GeneMarkS-2+) (10), and metrics reporting (QUAST v5.0.0) (11). Mash (v2.2.1; Mash Screen) (12) was used for taxonomic assignment of assemblies based on the highest RefSeq (v93; https://www.ncbi.nlm.nih.gov/refseq) identities that were >0.8 (excluding hits containing the words “phage” or “plasmid”) using reference 13 and www.ncbi.nlm.nih.gov/genome for Vibrio diabolicus classification. Analysis tools were used with default settings unless noted otherwise.

Isolate and genome details are listed in Table 1. Genome assemblies included 34 to 265 contigs and coverage depths of 23-fold to 180-fold. Total genome sizes ranged from 4.0 Mbp to 6.1 Mbp and contained 3,667 to 6,056 predicted genes. This resulted in draft *de novo* genomes for 38 Vibrio alginolyticus, 32 *V. diabolicus*, 10 V. parahaemolyticus, 1 V. cholerae, and 3 V. ordalii isolates and 1 unclassified *Vibrio* isolate.

### Data availability.

Data from this study have been deposited in NCBI under BioProject PRJNA839006; raw read SRA and GenBank accession numbers are listed in Table 1.
